# In Situ Composition and Thickness Monitoring during (Bi_x_In_1‑x_)_2_Se_3_ Thin Film Growth: toward Automated Synthesis Control Using Spectroscopic Ellipsometry for Quantum and Spintronic Devices

**DOI:** 10.1021/acsanm.6c00073

**Published:** 2026-04-06

**Authors:** Maria Hilse, Jackson Niedel, Qihua Zhang, Anthony Richardella, Hussein Hijazi, Jeffrey R. Shallenberger, Robert Hengstebeck, Stephanie Law, Nitin Samarth, Frank C. Peiris

**Affiliations:** † Two-Dimensional Crystal Consortium, Materials Innovation Platform, 8082 The Pennsylvania State University, University Park, Pennsylvania 16802, United States; ‡ Materials Research Institute, 8082 The Pennsylvania State University, University Park, Pennsylvania 16802, United States; § Department of Materials Science and Engineering, The Pennsylvania State University, University Park, Pennsylvania 16802, United States; ∥ Department of Physics, 3475 Kenyon College, Gambier, Ohio 43022, United States; ⊥ Department of Physics, Pennsylvania State University, University Park, Pennsylvania 16802, United States; # Physics Department, 242612 New Jersey Institute of Technology (NJIT), Newark, New Jersey 07102, United States; ∇ Institute of Energy and the Environment, Pennsylvania State University, University Park, Pennsylvania 16802, United States

**Keywords:** topological Insulators, molecular beam epitaxy, spectroscopic ellipsometry, dielectric functions, Bi_2_Se_3_, In_2_Se_3_

## Abstract

In this work, we show that by coupling *in situ* spectroscopic ellipsometry with a molecular beam epitaxy growth chamber, the growth parameters of ternary compounds with potential for quantum and spintronic applications can be immediately ascertained during the entire growth cycle of a sample. Initially, several films of (Bi_*x*_In_1–*x*_)_2_Se_3_ with stoichiometries ranging from *x* = 0 to *x* = 1 were grown and characterized by X-ray reflectivity, X-ray photoelectron spectroscopy, and Rutherford backscattering. Using this information, ellipsometry spectra were fitted by representing the dielectric functions with Kramers–Kronig-consistent oscillators. Consequently, composition-dependent dielectric functions of (Bi_*x*_In_1–*x*_)_2_Se_3_ were parametrized to create a material file that determines the Bi content of an unknown (Bi_*x*_In_1–*x*_)_2_Se_3_ film. By using this material file, therefore, both the Bi content and thickness of a (Bi_*x*_In_1–*x*_)_2_Se_3_ film can be obtained immediately at any stage of the growth cycle. We tested the model for universality among MBE growth systems and found that the model was transferable between systems. Furthermore, the generalized model allowed us to monitor sticking and desorption coefficients for Bi_2_Se_3_ thin films *in operando* for the first time, with significant implications for quantum and spintronic applications by enabling more reproducible and controlled device fabrication, advancing the understanding of emergent physics, and helping address future societal bottlenecks in electronic performance and demand.

## Graphical abstract

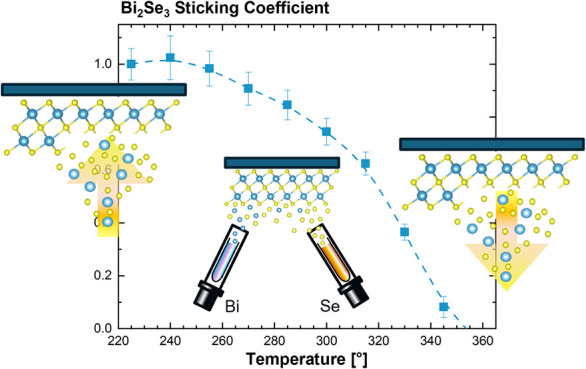

## 1 Introduction

In order to accommodate the growing need to fabricate novel and challenging structures serving as platforms for quantum and spintronic applications including topological insulators,^1,2^ transition metal dichalcogenides (TMDs),^3,4^ metal oxides,^5–7^ phosphorenes,^8–10^ MXenes,^11,12^ and Janus structures,^13–15^ it is important to develop *in situ* techniques that give instant feedback on composition and thickness during growth. While reflection high energy electron diffraction (RHEED) and laser interferometry techniques have been used to monitor growth in molecular beam epitaxy (MBE) and chemical vapor deposition (CVD) systems, these techniques are highly surface-sensitive and track foremost morphological changes.^16–20^ Both RHEED and laser interferometry provide only limited insight into thickness and compositional evolution, or yield signals that are difficult to interpret in practice, rendering them unsuitable for real-time growth monitoring in systems that do not follow a layer-by-layer growth mode, such as two-dimensional (2D) van der Waals materials.^21,22^ Spectroscopic ellipsometry (SE) is a nondestructive optical method that can be modeled to determine the dielectric function [ε­(*E)* = *ε*_1_ *+ iε*_2_] and the thickness of films. When coupled to growth chambers, SE produces immediate feedback on film thickness and composition.^23–25^ SE spectra can be obtained within seconds and analyzed at even shorter time scales with basic modern PCs, unlike transmission or reflectivity methods that require time-costly Kramers–Kronig transformations. *In situ* SE is therefore a promising and powerful tool for monitoring and optimizing growth conditions in real time for the fabrication of high-quality films and functional heterostructures.^26,27^ This is of particular interest when growing ternary or quaternary compounds as monitoring the dielectric function can ensure a specific alloy stoichiometry, which is challenging to achieve with conventional flux calibration methods since they are time-consuming and fluxes may drift during growth.

The investigation of Bi_2_Se_3_ and (Bi_*x*_In_1–*x*_)_2_Se_3_ thin film synthesis is crucial due to their unique properties as topological insulators, which hold promise for advanced quantum electronics and spintronic applications.^1,2^ Achieving precise control over stoichiometry and thickness during the growth process is essential, as these parameters directly influence the material’s electronic band structure and resulting functional properties. This work addresses the synthesis shortcomings by integrating *in situ* spectroscopic ellipsometry with molecular beam epitaxy, enabling immediate, nondestructive monitoring of both Bi content and thickness throughout the entire growth cycle. By parametrizing the dielectric functions based on Bi content and developing a transferable model, this approach enhances the ability to optimize growth conditions rapidly and reliably, thereby advancing the fabrication of high-quality Bi_2_Se_3_-based thin films for future device applications.

To properly set up an *in situ* SE feedback on Bi content and thickness for Bi–In–Se alloy thin film growth, *ε­(E)* must first be determined for a set of alloy samples, where the alloy Bi:In ratios are determined using other experimental techniques. The most integral part of this process is to perform SE measurements on pristine samples before they are taken out of the growth chamber. Oxidation and other contaminants that form postgrowth can influence the optical models used to obtain *ε­(E)* and to extract measurables like film thicknesses from SE spectra. Although several methods exist to model overlayers and other nonfilm components in *ex situ* SE, such methods compromise the precision and the accuracy of determining the correct *ε­(E)* of the film. The alloy growth process monitoring using *in situ* SE is sketched in Figure 1a where yellow arrows indicate the light path of the SE method.

**Figure 1:**
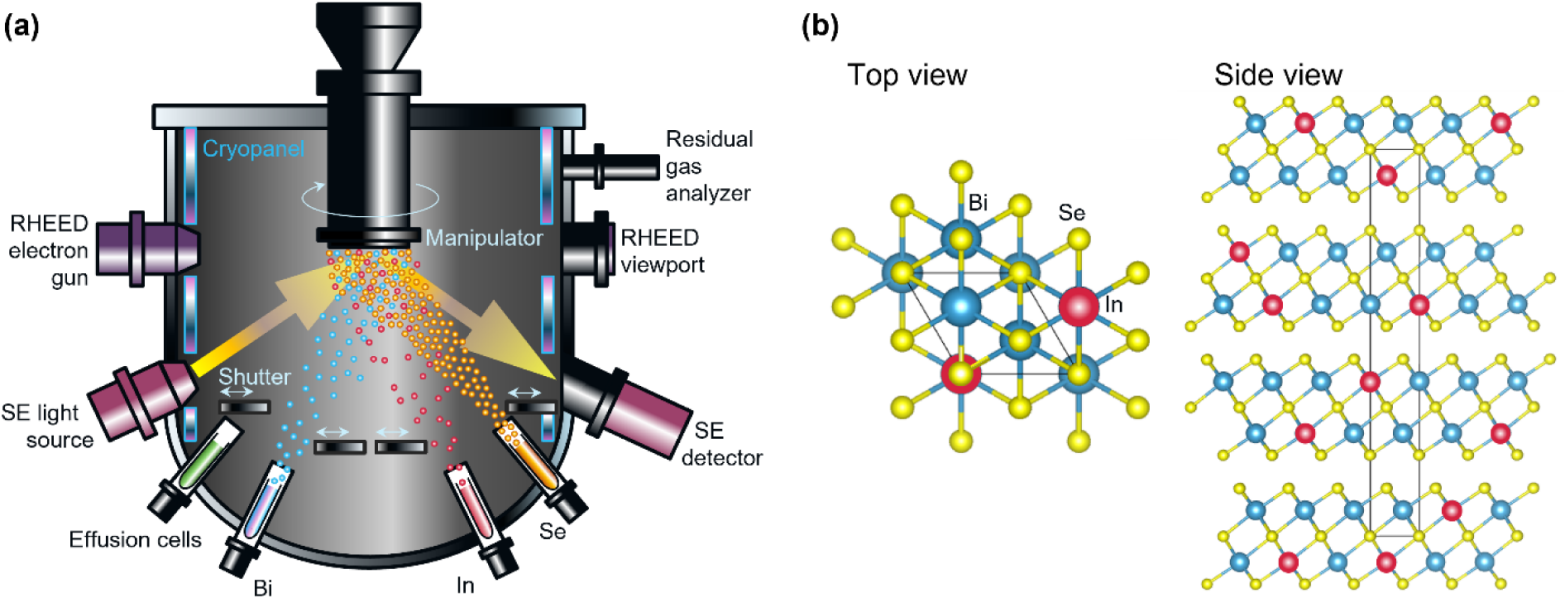
(a) Sketch of the MBE reactor in cross section demonstrating the Bi–In–Se alloy growth process and light path of the in situ SE measurements. (b) Arrangement of the Bi, In, and Se atoms in the targeted (Bi*_x_* In_1–*x*_)_2_Se_3_ crystal structure.

In this study, we have targeted (Bi_*x*_In_1–*x*_)_2_Se_3_ with the crystal structure as depicted in Figure 1b to show how *in situ* SE can be used to monitor the characteristics of thickness and Bi content of a sample during its entire growth cycle. Using a series of (Bi_*x*_In_1–*x*_)_2_Se_3_ samples with known thicknesses and known Bi contents ranging from *x* = 0, i.e., In_2_Se_3_ (100% In, 0% Bi) to *x* = 1, i.e., Bi_2_Se_3_ (0% In, 100% Bi), as determined through independent film characterization methods conducted *ex situ*, we determined their dielectric functions using SE. Based on this library of stoichiometry-dependent dielectric functions of (Bi_*x*_In_1–*x*_)_2_Se_3_, we can predict the alloy stoichiometry and the thickness of a (Bi_*x*_In_1–*x*_)_2_Se_3_ sample at any stage of its growth cycle using *in situ* SE. Having set up a reliable and precise *in situ* SE feedback, our study then shows how this can be used to determine temperature-dependent growth kinetics of (Bi_*x*_In_1–*x*_)_2_Se_3_ alloys, stoichiometry changes, desorption, and sticking coefficients observed solely through the *in situ* measurements.

## 2 Materials and Methods

MBE growth was carried out on single-side polished 3” diameter single crystal Al_2_O_3_(0001) wafers purchased from Cryscore with a 0.2° miscut along the [112̅0] direction. Before growth, wafers were first cleaned in a sequence of 3 min long ultrasonic baths of acetone, isopropyl alcohol, and DI water and blown dry with nitrogen. After solvent cleaning, a furnace anneal in air for 8 h at 1150 °C ensured the formation of atomic steps on the wafer surface with straight step edges. Wafers then went through a second sequence of 15 min long ultrasonic baths of acetone, isopropyl alcohol, and DI water to remove any furnace contamination. Wafers were finally mounted on ultrahigh vacuum (UHV) compatible sample holders and outgassed at 120 °C for 30 min in the load lock chamber of the MBE system. Thin film growth of (Bi_*x*_In_1–*x*_)_2_Se_3_ occurred in a DCA Instruments R450 MBE system (see DOI 10.60551/gqq8-yj90 for instrument details) at a background base pressure below 5 × 10^–10^ Torr. All SE calibration samples were grown following a two-step procedure where 3 quintuple layers (QL) were initially deposited at 135 °C after which the remaining film was deposited at 225 °C. Growth temperatures were monitored by thermocouple feedback on the backside of the wafer. The Se flux was supplied from a Veeco Mark V 500 cm^3^ valved Se cracker source operated at 320 °C in the bulk zone and 900 °C in the cracking zone and was kept constant at 2.6 × 10^–13^ atoms/(cm^2^s) for all samples. Bi and In fluxes were supplied by low and dual-temperature Knudsen effusion cells operated between 435 and 505 °C and 682–785 °C, respectively, generating fluxes in the range from 1.0 × 10^–12^ #/(cm^2^s) to 3.8 × 10^–13^ #/(cm^2^s). Flux calibration was done by inserting a quartz crystal microbalance (QCM) on a retractable arm into the sample position prior to film growth. Tooling factors for the QCM measurements were obtained from calibration growth runs and film thickness measurements using X-ray reflectivity (XRR). Reflection high energy electron diffraction (RHEED) was used during MBE growth to verify single crystal film formation.

*In situ* SE measurements were performed using a M-2000 *in situ* ellipsometer model X-210 (J.A. Woollam). Spectra were recorded in the range from 210 to 1690 nm with a fixed incident angle of 75°. Proper timing of the substrate rotation during growth ensured continuous SE monitoring during sample rotation. Routine calibration measurements of the SE were performed on a 25 nm-thick thermally grown native oxide layer on a Si wafer before the start of this project to ensure that the SE signal was not affected by unintentional changes in the optical pathway, e.g., deposition of Se on the optical viewports. After the sapphire substrates were transferred to the MBE reactor, a baseline SE measurement was taken of the Al_2_O_3_ substrate at the growth temperature.

After growth, XRR measurements were performed *ex situ* using a Malvern PANalytical 4-circle X’Pert^3^ X-ray diffractometer in line focus. Radiation generated from a Cu anode was directed onto the specimen through an incident beam slit of 1/32°, a parabolic mirror optic, and 10 mm mask. A PIXcel 3D detector in open detector mode with a 0.09° parallel plate collimator prefix was used for collecting the XRR data.

X-ray photoelectron spectroscopy (XPS) experiments were carried out on a Physical Electronics VersaProbe III instrument equipped with a monochromatic Al Kα X-ray source (hν = 1,486.6 eV) and a concentric hemispherical analyzer. Low energy electrons (<2 eV) and Ar ions (∼10 eV) were used to perform charge neutralization. Sputter cleaned Cu (Cu 2p_3/2_ = 932.62 eV, Cu 3p_3/2_ = 75.1 eV) and Au foils (Au 4f_7/2_ = 83.96 eV) were used to calibrate the binding energy axis.^28^ The selenide peak at 53.5 eV served as charge reference for peaks.^29^ The measurement geometry used a 45° takeoff angle with respect to the sample surface plane resulting in 95% of the signal originating from the top 3–6 nm of the film across a beam spot of ∼200 μm lateral size. Instrumental relative sensitivity factors (RSFs) that account for the X-ray cross section and inelastic mean free path of the electrons were used for signal quantification.

Rutherford backscattering (RBS) was performed with an energy resolution of 18 keV at the Rutgers Laboratory for Surface Modification Ion Scattering Facility using a focused ^4^He^2+^ ion beam of 2.3 MeV energy. The ion beam was directed onto the sample along the film normal and the backscattered beam was collected at 5 cm above the sample under 17° tilt from the film normal. RBS film stoichiometries were calculated based on the film densities determined from XRR.

## 3 Results and Discussion

To develop the SE model, we show in Figure 2 the XRR, RBS, XPS, and SE data from top to bottom obtained for three selected (Bi_*x*_In_1–*x*_)_2_Se_3_ alloys (Samples 11, 4, and 2 from left to right). The film thickness (d) was determined *ex situ* using XRR by fitting the data (green circles in Figure 2) using the AMASS software (red solid line in Figure 2). The thickness values are given in the XRR windows in Figure 2 for the respective samples. The film stoichiometry was measured *ex situ* using the two independent methods, RBS and XPS, as shown in the second and third lines of Figure 2. For each background-corrected RBS and XPS spectrum in green circles, the respective whole spectrum fit is given by a red solid line. The relative atomic percentages of Bi, In, and Se given by each peak were extracted from their individual contributions in the experimentally observed spectra shown as purple, blue, and green peaks, respectively, in the RBS and XPS data in Figure 2. The signal corresponding to Al and O from the substrate observed in RBS is indicated by pink and gray step functions, respectively. In the RBS data, slight deviations from the ideal 2:3 ratio of (Bi+In):Se range within the error margin of the method. The relative atomic percentages of Se measured by XPS in samples 4 and 2 as printed on the graph in Figure 2, row 3, column 2 and 3, respectively is significantly lower (59.1% and 55.3%) than the expected stoichiometric 60%. The discrepancy here does not indicate a high Se deficiency and nonstoichiometric films but originates from surface oxidation. Selenium oxides form foremost at the surface of noncapped films stored in ambient conditions that can dominate the Se signal from the film itself in the total measured XPS signal. Due to the high surface sensitivity and shallow penetration depth of the XPS method, even thin oxidation layers can skew the measured atomic percentage values significantly when measured on the surface only.

**Figure 2:**
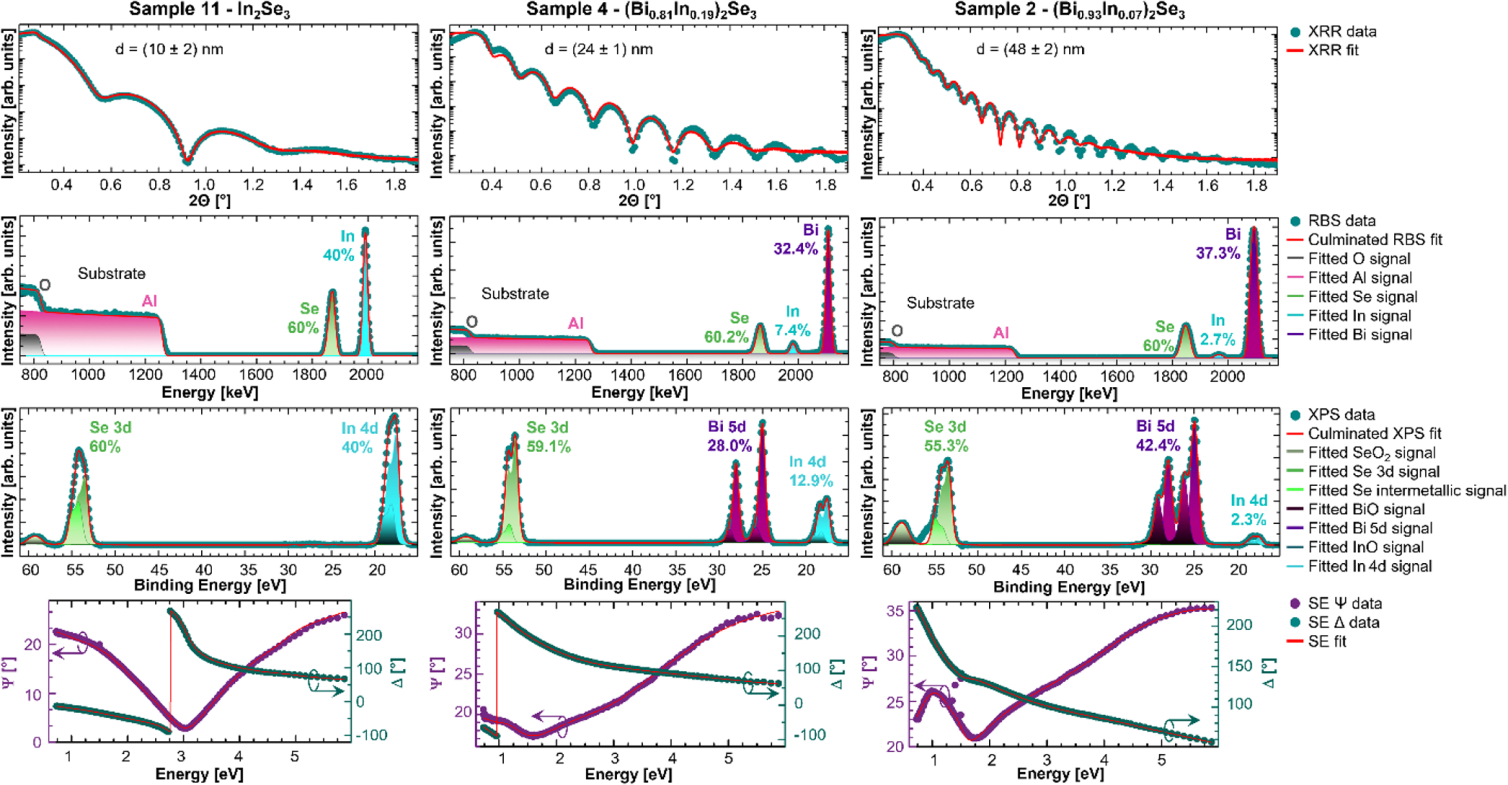
From top to bottom: XRR data with fit used to extract the layer thickness, d; RBS data with fitted individual elemental contributions and overall RBS fit; XPS data with fitted individual peaks and overall XPS fit; and the experimentally observed polarization angle Ψ and amplitude ratio Δ from *in situ* SE, together with the optical model fit, for representative samples 11, 4, and 2.

In addition to the example samples in Figure 2, 8 additional samples with varying Bi content were grown as the basis for the optical model development for *in situ* SE. The complete list of samples is summarized in Table 1 and include their Bi content *x*_RBS_ as measured by RBS, the Bi content *x*_XPS_ determined from XPS, and their measured film thickness (d) from XRR. (Sample 1, the pure Bi_2_Se_3_ film, was not measured by RBS, hence the blank in the *x*_RBS_ field.) Within the error margins, RBS and XPS give similar values for the stoichiometry of most ternary alloy samples as reflected by the Bi content *x*_RBS_ and *x*_XPS_ in Table 1. For Samples 4, 7, 8, and 9, the XPS method, however, reports lower Bi contents compared to RBS. This discrepancy likely arises from XPS’s high surface sensitivity and shallow penetration depth where even very thin oxidation layers can skew measured relative atomic percentages significantly similar to the mechanism behind underestimating the Se content from the ideal stoichiometric 60% value in XPS as discussed above. Under ambient conditions, Indium tends to oxidize preferentially at the surface, causing Indium to segregate near the top while Bismuth remains deeper below the oxide layer. As a result, XPS measures a smaller Bi content at the surface. In contrast, RBS probes the entire film, penetrating well into the substrate. Therefore, RBS provides the total film stoichiometry, allowing calculation of a more appropriate average Bi content that was used as baseline for modeling the ellipsometry data.

**Table 1:** Summary of the *ex situ* Determined Bi Content Measured by RBS (*x*_RBS_) and XPS (*x*_XPS_) in %, as Well as Film Thickness *d* in nm Determined through XRR Characterization for All Reference (Bi*_x_* In_1–*x*_)_2_Se_3_ Layers

	*x*_RBS_ in %	*x*_XPS_ in %	*d* in nm
Sample 1	-	100	29 ± 1
Sample 2	93 ± 3	94.9 ± 0.5	48 ± 2
Sample 3	89 ± 3	86.2 ± 0.7	55 ± 1
Sample 4	81 ± 3	68.5 ± 0.5	24 ± 2
Sample 5	77 ± 3	77.8 ± 0.2	22 ± 1
Sample 6	64 ± 3	62.7 ± 0.5	27.6 ± 0.1
Sample 7	47 ± 3	34.4 ± 0.3	37 ± 3
Sample 8	34 ± 3	23.6 ± 0.5	18 ± 2
Sample 9	18 ± 3	14 ± 1	31 ± 3
Sample 10	9 ± 3	5.7 ± 0.5	57 ± 3
Sample 11	0	0	14 ± 3

The experimental observables Ψ and Δ extracted from the SE data shown in Figure 2 as observed at the end of growth are a function of energy, angle of incidence, *ε­(E),* and the thickness of each layer in the sample. To determine the *ε­(E)* and the thickness of the (Bi_*x*_In_1–*x*_)_2_Se_3_ film, a two-layer model was developed, where the (Bi_*x*_In_1–*x*_)_2_Se_3_ layer was bound below by a semi-infinite sapphire substrate and above by vacuum. For the sapphire substrate, we used a previously determined birefringent *ε­(E)*, and the spectra were fitted with values of zero for all three Euler angles, confirming that the optical axis of sapphire was the film plane normal.^30,31^ By varying the model parameters, a regression analysis was performed using CompleteEase to match the calculated and experimental data, which produced *ε­(E)* and the thickness of the (Bi_*x*_In_1–*x*_)_2_Se_3_ layer. For most samples, fits were improved by incorporating a rough surface layer of around 0.3 nm thickness modeling the surface roughness of a real film. In order to model the experimental spectra, we represented the *ε­(E)* of (Bi_*x*_In_1–*x*_)_2_Se_3_ with appropriate Kramers–Kronig consistent oscillators, whose amplitude, energy-position and broadening parameters were adjusted to obtain the best fit, which is shown as solid lines in the Ψ and Δ spectra in Figure 2.

The corresponding real (*ε*_1_) and imaginary part (*ε*_2_) of *ε­(E)* for all (Bi_*x*_In_1–*x*_)_2_Se_3_ alloy samples are shown in Figure 3a and b, respectively. The maximum in *ε*_1_ shifts from around 3.0 eV continuously to lower energies while a distinct minimum around 3.5 eV develops with increasing Bi content. The imaginary part *ε*_2_ in Figure 3b, which corresponds to the absorption of the film, successively increases and the main absorption peak shifts from around 4.2 to 1.6 eV as the content of Bi increases in the alloy. More importantly, even small changes in the alloy stoichiometry cause sizable, consistent, and clearly conspicuous changes in *ε­(E)* (for example, see *x* = 93% in pink and *x* = 89% in purple in Figure 3) while the sample film thickness varies randomly. The fact that the imaginary part *ε*_2_ in Figure 3b is nonzero in some regions of the spectral range for samples with higher In content, indicates that these samples are not transparent in the spectral range of the measurement. Since the Ψ and Δ spectra are sensitive to both *ε­(E)* and thickness, these results suggest that even when the thickness is the same, samples that have small changes in the alloy Bi:In ratio will still show very distinct differences in the Ψ and Δ spectra. This in turn will enable simultaneous determination of the Bi content and thickness of a sample by obtaining *in situ* SE spectra. In general, the oscillators used to derive the dielectric functions correspond to electronic transitions, so analyzing their characteristics can provide insights into the material’s band structure. For example, as the In content increases, the zero-to-nonzero transition of ε_2_ shifts toward higher energies (blue shift), indicating an increase in band gap. This highlights the ability of this technique to track and analyze the electronic and topological properties of Bi–In–Se thin films in real time during their growth. Such monitoring is crucial for quantum and spintronic device applications, as it allows researchers to fine-tune material performance and ensure consistent reproducibility—both essential for the progress of quantum technologies. However, since this study focuses primarily on obtaining accurate dielectric functions, the detailed analysis of oscillator characteristics is not included in this work.

**Figure 3:**
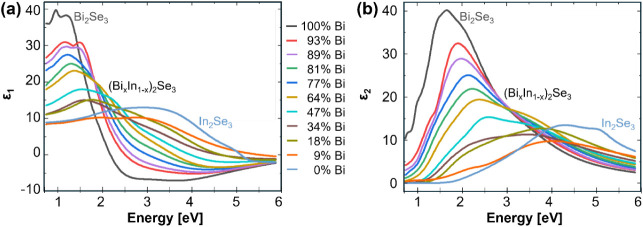
(a) Real ε_1_ and (b) imaginary part ε_2_ of the dielectric function obtained for the 11 (Bi*_x_* In_1–*x*_)_2_Se_3_ alloy samples as observed after growth labeled using the Bi content x in %.

Once we obtained the stoichiometry dependent *ε­(E)* for (Bi_*x*_In_1–*x*_)_2_Se_3_ alloys, we parametrized these functions to create a composite material file which can simulate the *ε­(E)* of any (Bi_*x*_In_1–*x*_)_2_Se_3_ alloy. With the material file, the *in situ* SE fit can accurately predict the Bi content and thickness of any unknown (Bi_*x*_In_1–*x*_)_2_Se_3_ film from a given Ψ and Δ spectra. The depth of information accessible from the *in situ* data that this material file unlocks is demonstrated in Figure 4 using sample 6 with a Bi content of 64% as an example. Selected snap shots of the Ψ and Δ spectra at different growth times are given starting from 0.04 min in Figure 4a all the way up to 20.04 min into growth in Figure 4f. While the *in situ* data collection records one full SE spectra every 7 s, in Figure 4, we show only select spectra for 6 representative times during the 20 min-long growth run. The solid lines in Figure 4 represent the fits that deliver alloy Bi content and thickness at each growth time interval. While *ex situ* experiments yield only one aggregate Bi content of the entire film, the *in situ* SE routine can determine the alloy Bi content and film thickness at any time during growth when data is collected.

**Figure 4:**
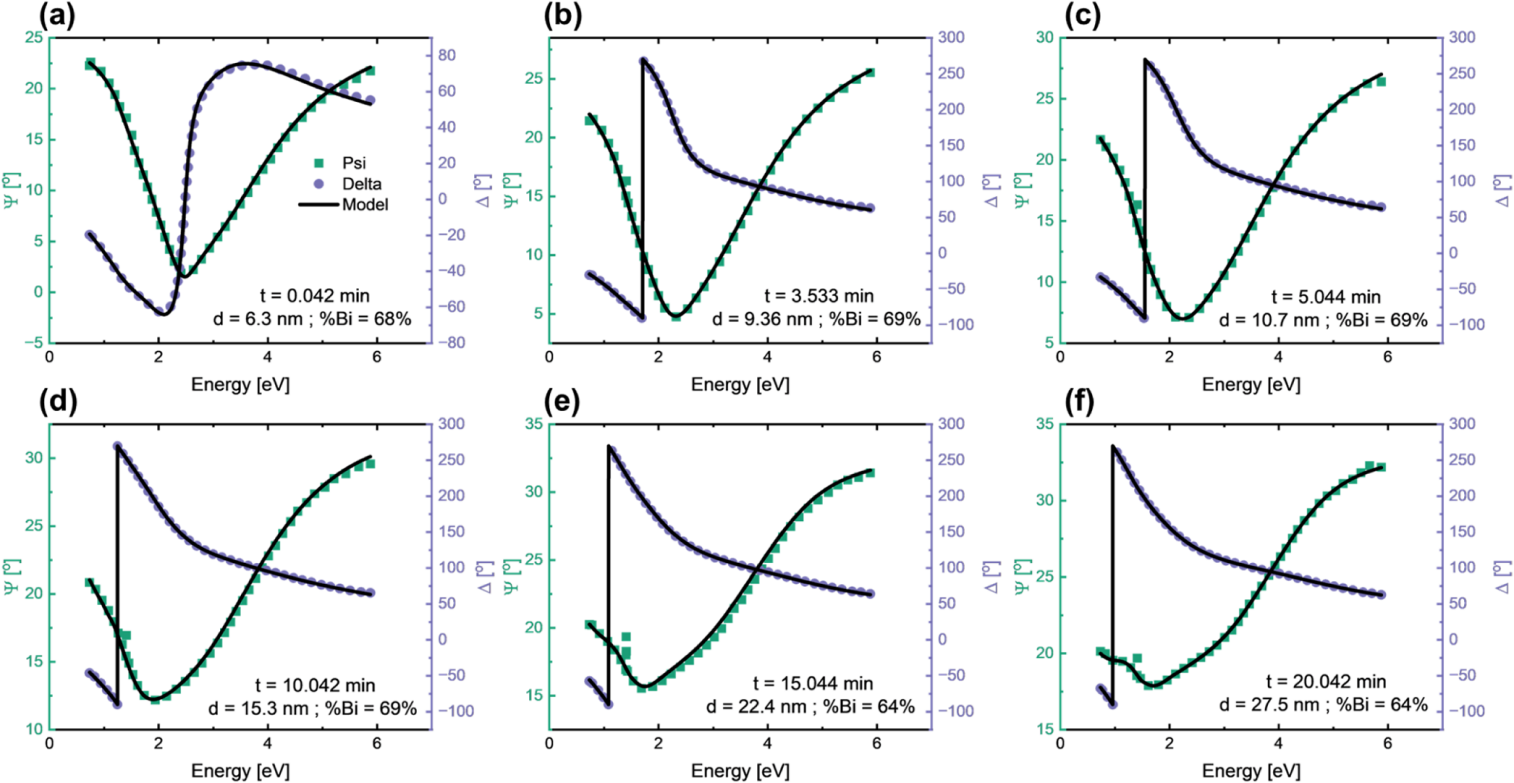
Spectra of ψ and Δ for a (Bi_0.64_In_0.36_)_2_Se_3_ film, i.e., sample 6 at different snap shots in growth time from (a) 0.04 min, (b) 3.53 min, (c) 5.04 min, (d) 10.04 min, (e) 15.04 min, to (f) 20.04 min into growth. Film thickness and Bi content extracted from the SE fit is given in each subfigure.

The film thickness and stoichiometry extracted using SE for all ternary reference samples is plotted in Figure 5 over the growth time with data points every 7 s. The data point density has been intentionally reduced in Figure 5 for better visibility. The full resolution of *in situ* SE is shown in the insets for sample 8 in Figure 5g, which presents the sample with the lowest growth rate of (0.06 ± 0.01) Å/s as measured by *in situ* SE and therefore highest resolution demonstrated in this work. The alloy Bi content and the thickness values for sample 6 (raw data in Figure 4) are shown in Figure 5e. For this sample, the RBS experiments gave a Bi content of 64%, and XPS characterization gave a Bi content of 63% in the top 3–6 nm of the film. The *in situ* SE method delivers growth time-dependent data revealing layer depth-dependent stoichiometry fluctuations showing up to 6% change in Bi content [the lowest and highest Bi contents were 63% and 70%, respectively with an average of (67 ± 2)% measured by *in situ* SE for sample 6]. SE results show that the thickness of all samples evolves nearly linearly over the growth time reflecting the constant growth rate. At first glance, the Bi content appears nearly constant over the growth time for most samples, as shown by the red circles in Figure 5, within a resolution limit of 0.1% as demonstrated by the smallest measured Bi content change in the inset of Figure 5g. It is important to note that this 0.1% resolution limit, combining instrumental and model uncertainties, is only achieved if the stoichiometry dependent *ε­(E)* for (Bi_*x*_In_1–*x*_)_2_Se_3_ alloys is known with a similar resolution. In our case, however, we have parametrized *ε­(E)* for (Bi_*x*_In_1–*x*_)_2_Se_3_ alloys by determining *ε­(E)* of only 11 samples, which in turn will increase the uncertainty of the stoichiometry. Indeed, the *in situ* SE method reveals slight fluctuations in the alloy stoichiometry in the first 20 min of growth for nearly all samples. The two exceptions are samples 4 and 5 in Figure 5c and d with Bi contents of (80.6 ± 0.1)% and (76.4 ± 0.5)%, respectively, reflected by the very small error margin in those samples where growth was stopped after 20 min. The Bi content was found to deviate by about 7% on average over all samples over the entire growth cycle with a maximum Bi content deviation of 17% for sample 7 compared to the Bi content measured at the end of the growth. A close look at Figure 5 shows changes in the film thickness corresponding to when fluctuations in the stoichiometry happen. Further work needs to be done to determine if these changes reflect fluctuations caused by real changes during growth (intermittent instabilities in Bi, Se, or In fluxes) or if these changes indicate instabilities in the *in situ* SE fitting routine. More in-depth SE modeling, possibly coupled with multimodel physics growth kinetics simulation, is required to provide further insight, which is beyond the presented study.

**Figure 5:**
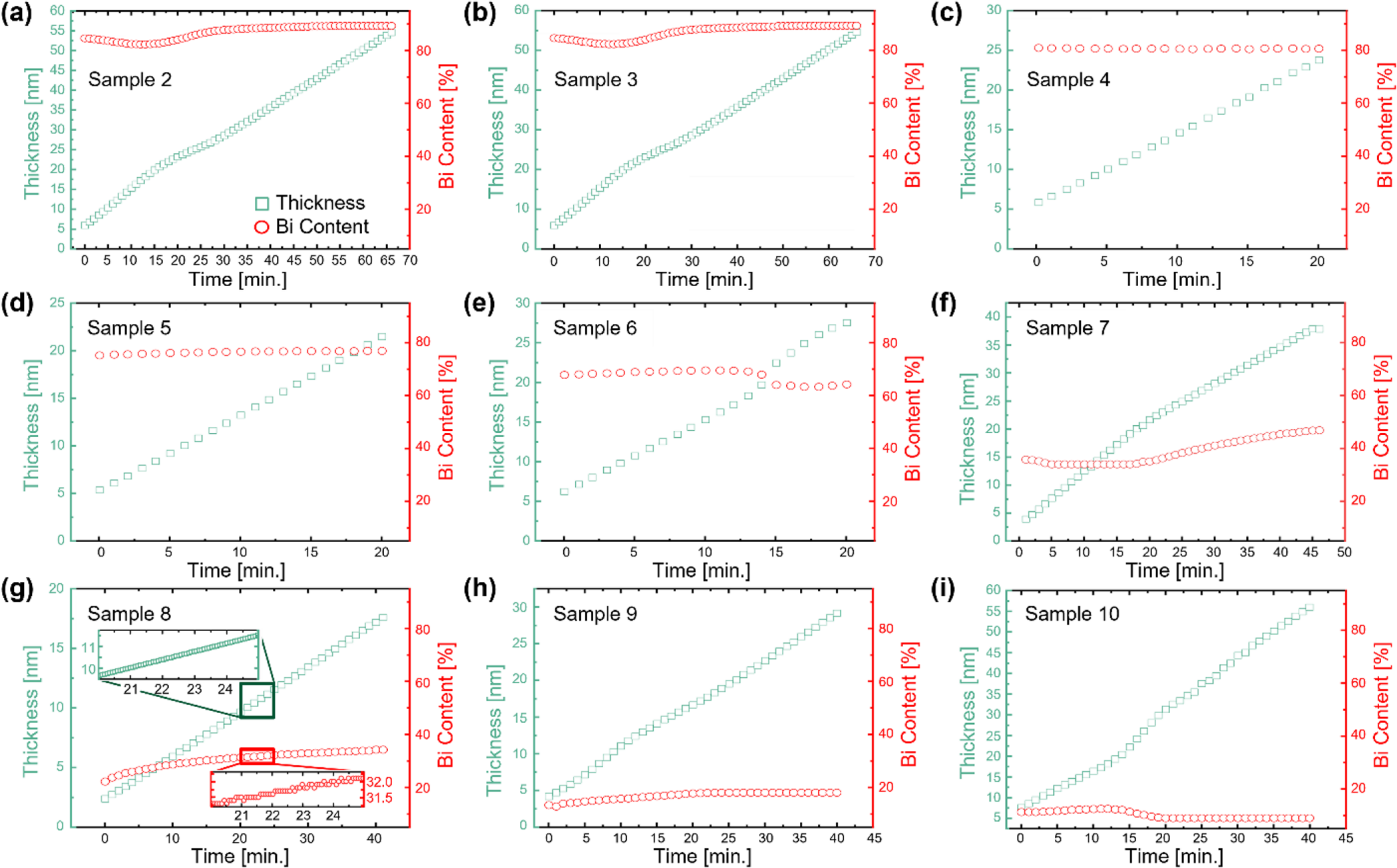
(a–i) show the Bi content and thickness measured by *in situ* SE over the growth time for the nine ternary (Bi*_x_* In_1–*x*_)_2_Se_3_ samples. The two insets in (g) show the full resolution of the *in situ* SE data equivalent to one full spectrum acquisition every 7 s for thickness and Bi content over the 5 min interval from 20 to 25 min of growth.

In addition to monitoring the alloy Bi content and the thickness during the growth of ternary samples, the *in situ* SE method can also record temperature-dependent growth dynamics. The temperature-dependent sticking and desorption coefficients for Bi_2_Se_3_ monitored *in situ* via the SE method are shown in Figure 6. To measure the sticking coefficient, a Bi_2_Se_3_ film of about 30 nm thickness was grown using typical growth conditions and a two-step growth procedure at 135 and 225 °C in a first step.^32^ The film growth rate was measured *in situ* by SE as change in film thickness per measurement interval. Since the measured growth rate at 135 and 225 °C was identical, this value was used as initial growth rate reference R_0_ assuming a sticking coefficient of 1 at both temperatures. The substrate temperature was then raised in increments. After temperature stabilization at each increment, Bi_2_Se_3_ growth was carried out for at least 10 min during which the new film growth rate at each temperature R_i_ was measured by *in situ* SE. The temperature-dependent sticking coefficient S was then calculated as S = R_0_/R_i_ shown in Figure 6a. The Bi_2_Se_3_ sticking coefficient starts to decrease noticeably at 270 °C, and decreases to nearly 50% at 320 °C. At temperatures of 350 °C and higher, a net zero Bi_2_Se_3_ growth rate was measured, indicating a sticking coefficient of zero. This behavior explains why low growth temperatures are required for Bi_2_Se_3_ thin film growth.^26,27,32,33^ To calculate the Bi_2_Se_3_ desorption coefficient, a similar film of Bi_2_Se_3_ with a thickness *d*_0_ as measured by *in situ* SE, was grown under the typical growth conditions. The film thickness *d*(*T*) was then measured continuously with *in situ* SE as the film was slowly heated with the Bi cell shutter closed (Se was supplied for the entire desorption process) until the measured film thickness was zero. This cycle was done twice with different heating rates of 5 °C/min and 10 °C/min. The desorption coefficient *D* was then calculated as *D* = 1 – *d*(*T*)/*d*_0_ shown in Figure 6b. The temperature region where the sticking coefficient reaches zero (340–360 °C) is highlighted by a light blue bar in Figure 6b and circled in Figure 6a with an arrow connecting both regions for ease of comparison. In this temperature range, the onset of film desorption was observed with a small desorption coefficient, barely rising to 10% for both the slower and faster heating rate. The Bi_2_Se_3_ films were eventually fully desorbed at 460 °C. Fitting the desorption coefficient data in an Arrhenius plot yields an activation energy *E*_a_ = (1.46 ± 0.04) eV for Bi_2_Se_3_ thin film desorption. It should be noted that the optical model assumed the presence of an intact Bi_2_Se_3_ film rather than a Bi-rich layer remaining on the substrate during the desorption and sticking coefficient measurements. However, the well-established difference in volatility between Bi and Se can lead to the formation of nonstoichiometric, Bi-rich films at elevated growth or desorption temperatures.^34^ While the assumption of stoichiometric Bi_2_Se_3_ is supported by the satisfactory fits obtained for spectra recorded below 375 °C, the fit quality deteriorates at higher temperatures, suggesting changes in the composition or morphology of the remaining film. To address this possibility, we extended the optical model to include a composite layer consisting of Bi_2_Se_3_ and Bi with variable constituent concentrations.^35^ However, this approach did not yield improved χ2 values. Similarly, increasing the roughness of the remaining Bi_2_Se_3_ layer did not improve the fits. Consequently, in the present work we assume that the remaining film consists entirely of Bi_2_Se_3_, while more systematic studies will be required in the future to elucidate the nature of the residual film at temperatures above 375 °C.

**Figure 6:**
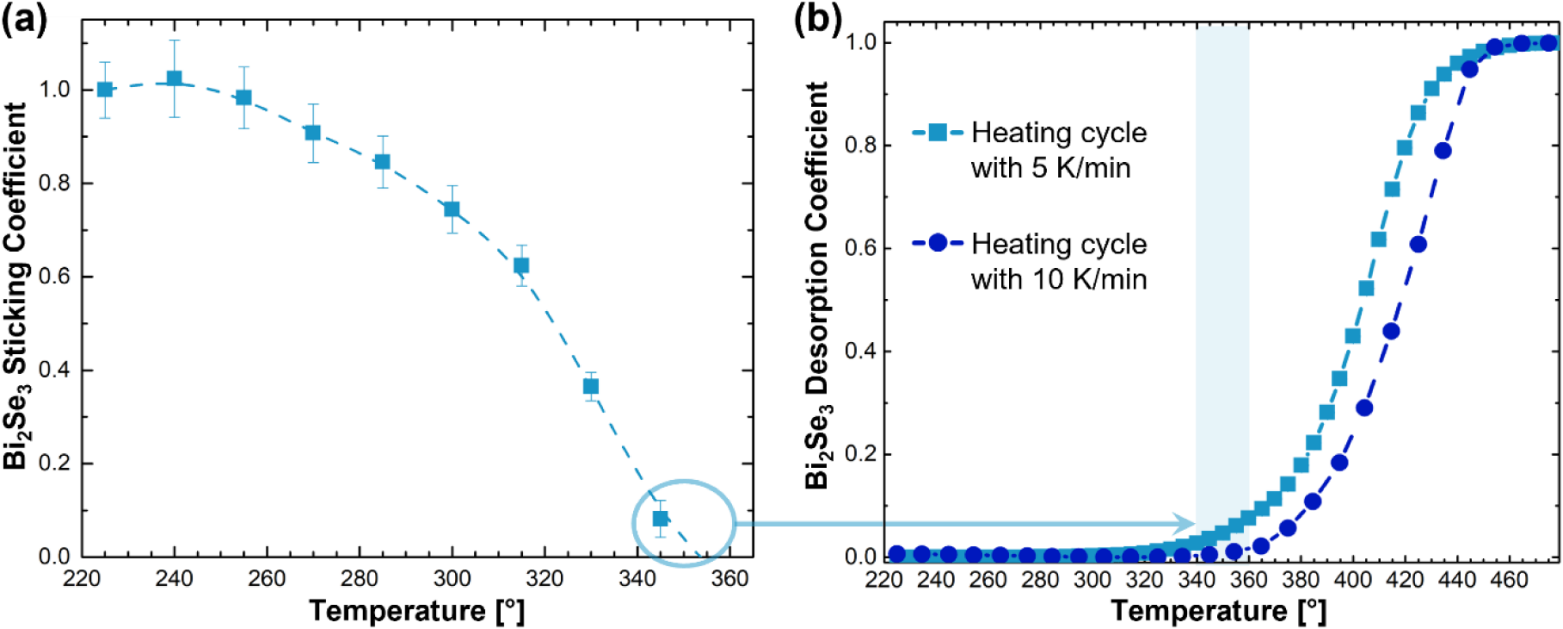
(a) Temperature-dependent sticking coefficient and (b) desorption coefficient measured *in situ* using spectroscopic ellipsometry for Bi_2_Se_3_ thin film growth on Al_2_O_3_(0001). The dashed lines connecting the data points represent a guide to the eye.

Figure 7 shows the optical model, that was developed on one MBE system – MBE #1 – applied to *in situ* film thickness monitoring at a different MBE system – MBE #2. To maximize the contrast between the two chosen MBE chambers, MBE #2 was of different make and model (multimodule characterization cluster MBE system manufactured by ScientaOmicron) where Bi_2_Se_3_ was grown using a valved Se cracker source instead of a simple Knudsen effusion cell. Further instrument details about MBE #2 can be found under DOI 10.60551/jb0f-5784. As Bi_2_Se_3_ thin films are relatively easy to grow by MBE, the films produced in both systems show streaky RHEED images indicative of long-range crystallographic order and smooth film surfaces as shown in Figure 7a. XRR data obtained *ex situ* for both films is shown in Figure 7b with the fitted curves reflecting the different growth rates and growth times used on both systems yielding total film thicknesses of (29 ± 1) nm and (15 ± 1) nm for the films grown in MBE #1 and MBE #2, respectively. As demonstrated by the smooth linear line of film thickness measured over the growth time, the *in situ* SE monitoring technique can monitor the Bi_2_Se_3_ growth process accurately across the two different systems using the same optical model. The final film thicknesses measured by *in situ* SE on both MBE systems are in good agreement with the results found by XRR. The model and technique is therefore easily transferable as it yielded accurate growth monitoring results across two entirely different synthesis reactors.

**Figure 7:**
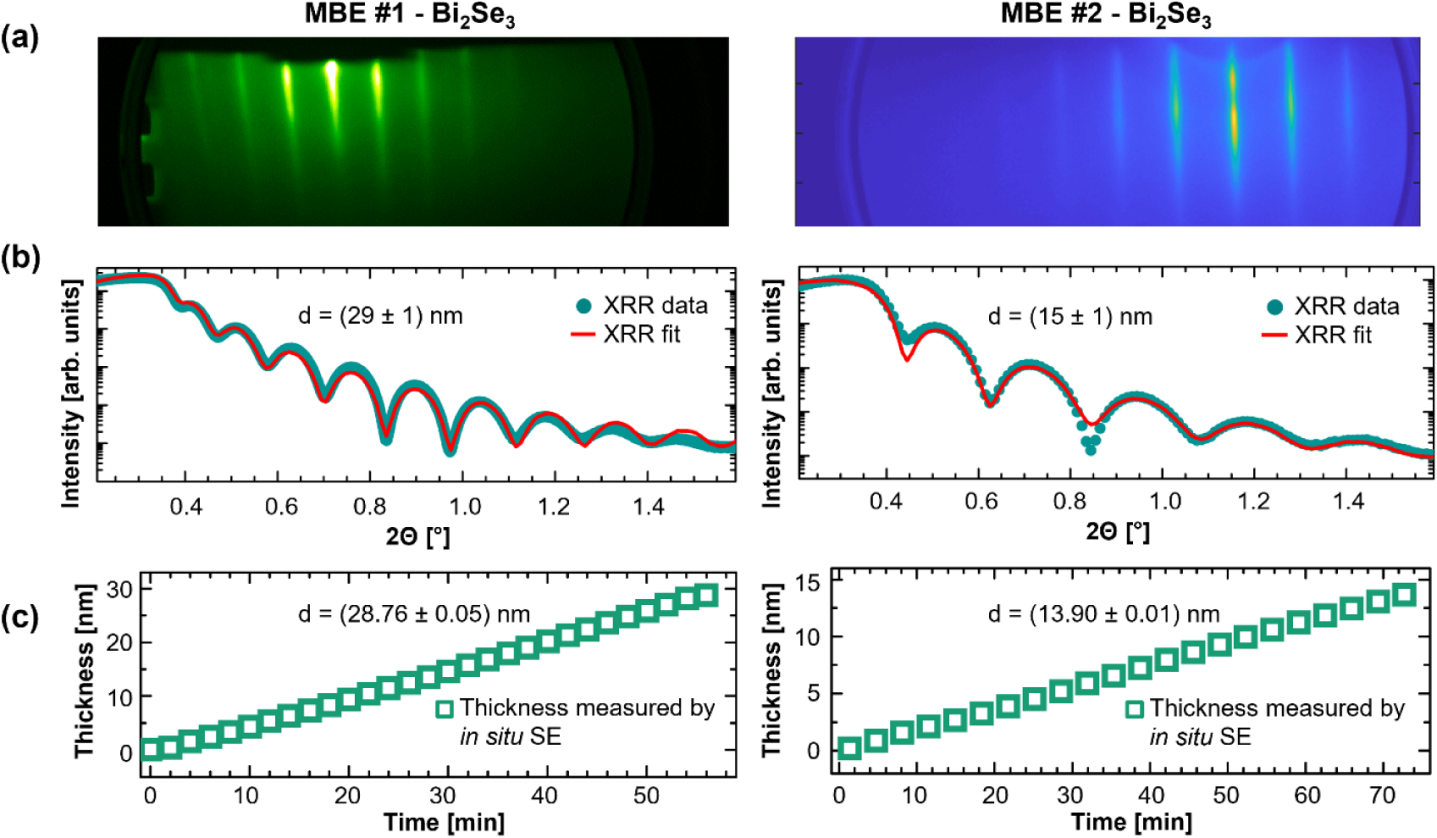
(a) RHEED images and (b) XRR data including the fitted curve yielding the total grown Bi_2_Se_3_ film thickness d as printed in each graph as well as (c) the film thickness measured using *in situ* SE over the entire growth time obtained from growths of Bi_2_Se_3_ thin films on Al_2_O_3_(0001) in two different MBE systems: MBE #1 and MBE #2.

## 4 Conclusion

In summary, this work demonstrates the effectiveness and versatility of our newly developed optical model for *in situ* SE monitoring of the growth dynamics, Bi content in the alloy, and film thicknesses of binary and ternary (Bi_*x*_In_1–*x*_)_2_Se_3_ thin films with high temporal resolution. By parametrizing the dielectric function and creating a transferable material file, our study enabled accurate, real-time tracking of growth parameters across different MBE systems. The method provided direct insights into the temperature-dependent sticking and desorption coefficients of Bi_2_Se_3_ for the first time, revealing key aspects of the growth kinetics and offering a means to optimize synthesis conditions in real time. The optical model development for *in situ* SE represents a significant step toward the precise, reproducible, and automated fabrication of complex chalcogenide thin films, paving the way for advancing research in topological insulators and related materials for quantum and spintronic applications.

## Data Availability

All data of this study is freely available on ScholarSphere under the following link: https://doi.org/10.26207/rpw6-3n35.

## References

[1] Moore J. E. (2010). The Birth of Topological Insulators. Nature.

[2] Hasan M. Z., Kane C. L. (2010). Colloquium: Topological Insulators. Rev. Mod. Phys..

[3] Manzeli S., Ovchinnikov D., Pasquier D., Yazyev O. V., Kis A. (2017). 2D Transition Metal Dichalcogenides. Nat. Rev. Mater..

[4] Mak K. F., Lee C., Hone J., Shan J., Heinz T. F. (2010). Atomically Thin MoS 2: A New Direct-Gap Semiconductor. Phys. Rev. Lett..

[5] Xie H., Li Z., Cheng L., Haidry A. A., Tao J., Xu Y., Xu K., Ou J. Z. (2022). Recent Advances in the Fabrication of 2D Metal Oxides. iScience.

[6] Hu X., Liu K., Cai Y., Zang S.-Q., Zhai T. (2022). 2D Oxides for Electronics and Optoelectronics. Small Sci..

[7] Guan M., Wang Q., Zhang X., Bao J., Gong X., Liu Y. (2020). Two-Dimensional Transition Metal Oxide and Hydroxide-Based Hierarchical Architectures for Advanced Supercapacitor Materials. Front. Chem..

[8] Carvalho A., Wang M., Zhu X., Rodin A. S., Su H., Castro Neto A. H. (2016). Phosphorene: From Theory to Applications. Nat. Rev. Mater..

[9] Akhtar M., Anderson G., Zhao R., Alruqi A., Mroczkowska J. E., Sumanasekera G., Jasinski J. B. (2017). Recent Advances in Synthesis, Properties, and Applications of Phosphorene. Npj 2D Mater. Appl..

[10] Goswami A., Gawande M. B. (2019). Phosphorene: Current Status, Challenges and Opportunities. Front. Chem. Sci. Eng..

[11] Gogotsi Y., Anasori B. (2019). The Rise of MXenes. ACS Nano.

[12] Shamsabadi A. A., Fang H., Zhang D., Thakur A., Chen C. Y., Zhang A., Wang H., Anasori B., Soroush M., Gogotsi Y. (2023). The Evolution of MXenes Conductivity and Optical Properties Upon Heating in Air. Small Methods.

[13] Petrić M. M., Kremser M., Barbone M., Qin Y., Sayyad Y., Shen Y., Tongay S., Finley J. J., Botello-Méndez A. R., Müller K. (2021). Raman Spectrum of Janus Transition Metal Dichalcogenide Monolayers WSSe and MoSSe. Phys. Rev. B.

[14] Zhang L., Xia Y., Li X., Li L., Fu X., Cheng J., Pan R. (2022). Janus Two-Dimensional Transition Metal Dichalcogenides. J. Appl. Phys..

[15] Ju L., Bie M., Shang J., Tang X., Kou L. (2020). Janus Transition Metal Dichalcogenides: A Superior Platform for Photocatalytic Water Splitting. J. Phys.: Mater..

[16] Maksym P. A., Beeby J. L. A. (1981). Theory of Rheed. Surf. Sci..

[17] Hafez M. A., Zayed M. K., Elsayed-Ali H. E. (2022). Review: Geometric Interpretation of Reflection and Transmission RHEED Patterns. Micron.

[18] Boebel, F. G. ; M?ller, H. ; Preib, W. Reflexion Supported Pyrometric Interferometry: Anew Tool for in Situ, Real Time Temperature Control in Semiconductor Manufacturing Proceedings. IEEE/SEMI Advanced Semiconductor Manufacturing Conference and Workshop IEEE boston, MA. 1993 130–134 10.1109/ASMC.1993.682496

[19] Boebel F. G., Möller H., Hertel B., Grothe H., Schraud G., Schröder S., Chow P. (1995). In Situ Film Thickness and Temperature Control of Molecular Beam Epitaxy Growth by Pyrometric Interferometry. J. Cryst. Growth.

[20] Boebel, F. G. ; Moller, H. Simultaneous in Situ Measurement of Film Thickness and Temperature by Using Multiple Wavelengths Pyrometric Interferometry (MWPI) IEEE/SEMI Conference and Workshop on Advanced Semiconductor Manufacturing IEEE 1993 6 2 112–118 10.1109/66.216929

[21] Chandril S., Keenan C., Myers T. H., Lederman D. (2009). In Situ Thin Film and Multilayer Structural Characterization Using x-Ray Fluorescence Induced by Reflection High Energy Electron Diffraction. J. Appl. Phys..

[22] Sun H. Y., Mao Z. W., Zhang T. W., Han L., Zhang T. T., Cai X. B., Guo X., Li Y. F., Zang Y. P., Guo W., Song J. H., Ji D. X., Gu C. Y., Tang C., Gu Z. B., Wang N., Zhu Y., Schlom D. G., Nie Y. F., Pan X. Q. (2018). Chemically Specific Termination Control of Oxide Interfaces via Layer-by-Layer Mean Inner Potential Engineering. Nat. Commun..

[23] Daraselia M., Brill G., Garland J. W., Nathan V., Sivananthan S. (2000). In-situ control of temperature and alloy composition of Cd1–xZnxTe grown by molecular beam epitaxy. J. Electron. Mater..

[24] Hilfiker, J. N. In Situ Spectroscopic Ellipsometry (SE) for Characterization of Thin Film Growth. In In Situ Characterization of Thin Film Growth; Elsevier Ltd: Cambridge, 2011; pp 99–151. DOI: 10.1533/9780857094957.2.99.

[25] Yoshikawa A., Xu K., Taniyasu Y., Takahashi K. (2002). Spectroscopic Ellipsometry In-Situ Monitoring/Control of GaN Epitaxial Growth in MBE and MOVPE. Phys. Stat. Sol..

[26] Hilse M., Wang X., Killea P., Peiris F., Engel-Herbert R. (2021). Spectroscopic Ellipsometry as an In-Situ Monitoring Tool for Bi2Se3 Films Grown by Molecular Beam Epitaxy. J. Cryst. Growth.

[27] Bai A., Hilse M., Patil P. D., Engel-Herbet R., Peiris F. (2022). Probing the Growth Quality of Molecular Beam Epitaxy-Grown Bi2Se3 Films via in-Situ Spectroscopic Ellipsometry. J. Cryst. Growth.

[28] Seah M. P. (2001). Summary of ISO/TC 201 Standard: VII ISO 15472: 2001—Surface Chemical Analysis—X-ray Photoelectron Spectrometers—Calibration of Energy Scales. Surf. Interface Anal..

[29] Golyashov V. A., Kokh K. A., Makarenko S. V., Romanyuk K. N., Prosvirin I. P., Kalinkin A. V., Tereshchenko O. E., Kozhukhov A. S. (2012). Inertness and Degradation of (0001) Surface of Bi_2_Se_3_ Topological Insulator. J. Appl. Phys..

[30] Malitson I. H. (1962). Refraction and Dispersion of Synthetic Sapphire. J. Opt. Soc. Am..

[31] Schmidt D. (2014). Characterization of Highly Anisotropic Three-Dimensionally Nanostructured Surfaces. Thin Solid Films.

[32] Koirala N., Brahlek M., Salehi M., Wu L., Dai J., Waugh J., Nummy T., Han M. G., Moon J., Zhu Y., Dessau D., Wu W., Armitage N. P., Oh S. (2015). Record Surface State Mobility and Quantum Hall Effect in Topological Insulator Thin Films via Interface Engineering. Nano Lett..

[33] Zhang Q., Ou Y., Hilse M., Liu D. S. H., Law S. (2025). Prospects for THz Optoelectronic Devices Using Chalcogenide Topological Materials and Recent Progress on Their Synthesis by Molecular Beam Epitaxy. Opt. Mater. Express..

[34] Cafaro M. L., Bardi G., Piacente V. (1984). Vaporization Study of Solid Bismuth Selenide (Bi_2_Se_3_). J. Chem. Eng. Data.

[35] Alexandre B. S. C., Martins L. C., Santos J. E., Pontes A. J., Peres N. M. R. (2020). Fresnel Polarisation of Infra-Red Radiation by Elemental Bismuth. Eur. Phys. J. B.

